# Reconstruction After Wide Excision of the Nail Apparatus in the Treatment of Melanoma: A Systematic Literature Review

**DOI:** 10.3390/jcm14175932

**Published:** 2025-08-22

**Authors:** Luc Chouquet, Feriel Boukari, Thierry Balaguer, Henri Montaudié, Olivier Camuzard, Elise Lupon

**Affiliations:** 1 Plastic Surgery Department, Institut Universitaire Locomoteur et du Sport, Pasteur 2 Hospital, University Côte d’Azur, 06100 Nice, France; chouquet.l@chu-nice.fr (L.C.); balaguer.t@chu-nice.fr (T.B.); camuzard.o@chu-nice.fr (O.C.); 2 Dermatology Department, L’Archet Hospital, University Côte d’Azur, 06100 Nice, France; boukari.f@chu-nice.fr (F.B.); montaudie.h@chu-nice.fr (H.M.); 3 Laboratory of Molecular PhysioMedicine (LP2M), CNRS, University Côte d’Azur, 06100 Nice, France

**Keywords:** subungual melanoma, nail reconstruction, wide local excision, nail apparatus, nail melanoma

## Abstract

**Background/Objectives**: Historically, the treatment of subungual melanoma was based on amputation of the affected digit. However, extended wide local excision of the nail apparatus is now considered the conservative gold standard for in situ or minimally invasive forms. There are many after wide local excision reconstruction techniques, but few studies have objectively compared their results. The objectives were to carry out a systematic review of reconstruction after wide local excision reconstructions in the treatment of subungual melanoma. **Methods:** This systematic review was conducted following the PRISMA (Preferred Reporting Items for Systematic Reviews and Meta-Analyses) guidelines. An exhaustive search was conducted in the PubMed (Medline), Embase, and Cochrane Library databases, up to July 2025. Articles reporting reconstructions after wide local excision for subungual melanoma of the fingers or toes were included. Clinical, technical, and outcome data were analyzed. **Results:** The literature review comprised 24 articles on 373 patients, primarily those with in situ subungual melanoma. Reconstruction was most often performed using total skin grafts, sometimes combined with dermal matrices. Some authors used local or free flaps. Few studies used validated functional scores. Local recurrences were significant, affecting 18% of patients and requiring secondary amputation. **Conclusions:** Nail apparatus reconstructions are primarily indicated for in situ or minimally invasive subungual melanomas. Immediate reconstruction carries a risk of performing the reconstruction over residual tumor tissue, particularly in the case of invasive melanomas. Reconstructive techniques, such as full-thickness skin grafts and the use of dermal matrices, can provide satisfactory functional and aesthetic outcomes. However, objective evaluations of these results remain limited, and better standardization of clinical practice, along with prospective studies, is needed to refine long-term outcome assessment.

## 1. Introduction

Melanoma is a significant public health concern, with over 330,000 new cases and more than 58,000 deaths per year worldwide by 2022 [1]. One of the major prognostic factors for cutaneous melanoma is the Breslow index, which corresponds to the measured vertical thickness of the tumor, expressed in millimeters, from the top of the granular layer of the epidermis (or the base of an ulcer, if present) to the deepest point of tumor invasion in the dermis or subcutis. A greater Breslow thickness is strongly associated with a higher risk of recurrence and poorer survival outcomes [2,3]. However, subsequent genomic studies have suggested that this paradigm may not fully apply to nail apparatus melanoma, which appears genetically distinct from acral and other cutaneous melanomas, potentially influencing prognostic interpretation [4]. Subungual melanomas (SUM) belong to the subcategory of acral-lentiginous melanomas, which represent approximately 0.7 to 3.5% of all melanomas in Caucasians but can be as high as 50% in Asian or African Caribbean subjects [5,6]. SUM is most frequently found in the nail matrix, where it manifests as a dark, heterochromatic, longitudinal melanonychia with ill-defined contours that can sometimes invade adjacent skin folds. Historically, treatment was based on systematic amputation of the affected digit, often resulting in significant functional loss and cosmetic damage, which had a considerable impact on quality of life [7]. Today, it has been established that in situ or minimally invasive melanomas can be treated conservatively, by wide local excision (WLE) of the nail apparatus [8,9,10]. SUM involves excision of the entire nail with margins generally between 5 and 10 mm, and immediate or delayed reconstruction [11]. The choice of reconstruction technique is complex and not consensual, particularly when the excision is large, local functional requirements are essential, and scarring threatens to cause cosmetic damage with social repercussions [12,13].

To better identify the possibility of reconstruction, we conducted a systematic review of the literature on surgical techniques used in nail reconstruction following SUM.

The objective of this review was to systematically evaluate the oncologic, functional, and aesthetic outcomes of conservative versus amputation surgical techniques for subungual melanoma, focusing on recurrence rates, reconstruction methods, and long-term quality-of-life impact.

## 2. Materials and Methods

- Literature search

This systematic review was conducted following the PRISMA (Preferred Reporting Items for Systematic Reviews and Meta-Analyses) guidelines [14]. An exhaustive search was conducted in PubMed/MEDLINE, Embase, Scopus, and Web of Science from their creation to July 2025. The keywords used were (‘Nail apparatus Melanoma’ OR ‘Nail Matrix Melanoma’ OR ‘Subungual Melanoma’) AND (‘reconstruction’ OR “flap” OR ‘dermal matrix’ OR ‘skin graft’ OR ‘healing’). Searches in Scopus and Web of Science did not identify additional eligible articles beyond those retrieved from PubMed and Embase. A manual search for the bibliographies of relevant articles completed the analysis. Only articles in English, Spanish, and French were retained. After an initial screening of titles and abstracts, duplicates, irrelevant studies, literature reviews, animal studies, and cases of amputation were excluded. The full texts were then analyzed, and purely anatomical studies were excluded. The institutional ethics committee has approved this research project.

- Study Selection

Studies were included according to the following criteria:

- Type of study: case series, case reports, cohort studies, and randomized controlled trials of nail reconstruction after melanoma removal.
- Participants: adult or pediatric patients undergoing reconstruction after removal of a subungual melanoma of the upper or lower limb.
- Results reported: type of melanoma and excision (margins), reconstruction technique, time frame, adjuvant treatments, complications, functional and aesthetic results, patient satisfaction, recurrences, and length of follow-up.

Two reviewers (L.C. and E.L.) independently examined the titles and abstracts and then the full texts of potentially eligible articles. Disagreements were resolved by discussion or arbitration by a third reviewer (O.C.).

- Data Collection

Due to the heterogeneity of the studies (in terms of design, population, and techniques), a meta-analysis was not feasible. A narrative summary was produced, and the results were presented in the form of summary tables, which brought together the main characteristics and conclusions of the included studies. Data were independently extracted by two reviewers using a standardized data collection form. Extracted variables included study design, population characteristics, surgical technique, follow-up duration, recurrence, functional and aesthetic outcomes, and complications.

- Data synthesis and analysis

Given the heterogeneity of study designs and outcome measures, a meta-analysis was not preferred. Instead, a narrative synthesis of the included studies was carried out. The data were organized and summarized in tabular form, highlighting the characteristics of the included studies, key findings, and outcomes of interest. In addition to extracting clinical and technical variables, we assessed the methodological quality of each included study. Risk of bias was evaluated independently by two reviewers using the Newcastle–Ottawa Scale (NOS) for observational studies and the Joanna Briggs Institute (JBI) checklists for case series or single case reports; disagreements were resolved by consensus. For each study, we also recorded the method used to assess functional and/or aesthetic outcomes, specifying whether validated patient-reported outcome measures (PROMs) were employed.

## 3. Results

- Study selection

**Figure 1:**
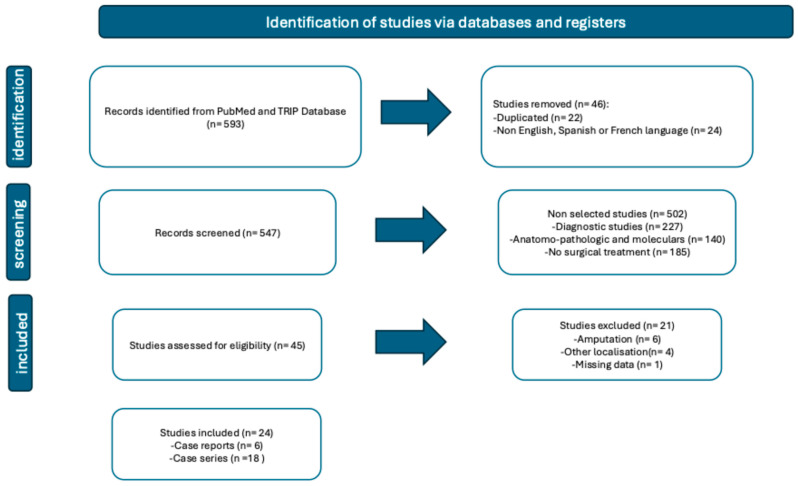
Flowchart.

The study selection process is summarized in the PRISMA 2020 flow diagram (Figure 1). An initial search conducted in the PubMed and TRIP databases identified 593 articles. After removing duplicates and excluding articles written in languages other than French, English, or Spanish, 547 unique references were included for screening. Title and abstract review led to the selection of 45 articles for full-text assessment. Of these, 21 were excluded: 16 focused on cases managed by amputation, 1 provided insufficient data, and 4 dealt with acral lentiginous melanomas at unrelated anatomical sites. In total, 24 studies met the predefined inclusion and exclusion criteria and were included in the analysis (Figure 1).

- Study characteristics

Of the 24 studies selected, 18 were case series [15,16,17,18,19,20,21,22,23,24,25,26,27,28,29,30,31,32], and 6 were single case reports [33,34,35,36,37,38]. All the studies were retrospective (*n* = 23), except for one study, which was a prospective case series [38]. All the studies were published between 2003, for the oldest [19], and 2023, for the most recent [22]. Table 1 summarizes the characteristics of the included studies.

Quality assessment revealed an overall moderate methodological level across the included studies. Most clearly stated their inclusion criteria and surgical protocols, but sample sizes were generally small, and comparative designs were rare. According to the Newcastle–Ottawa Scale (NOS) and Joanna Briggs Institute (JBI) checklist, depending on study design, methodological quality was rated as moderate in 13 studies, moderate-to-high in 8, and high in 3. Only one study [24] employed psychometrically validated patient-reported outcome measures (QuickDASH and Foot Function Index); all others relied on non-validated Likert-type or clinical scales for functional and aesthetic evaluation. Outcome assessment was heterogeneous, most often based on qualitative surgeon or patient appraisal (e.g., overall satisfaction, pain reports, or return to daily activities). Full details of the quality appraisal, including individual study ratings and notes on outcomes, are available in Supplementary Table S1.

- Demographic characteristics

Sample sizes varied from study to study, ranging from 1 to 140 [22] participants, for a total of 373 patients, with an average age of 53.4 years (4 to 81 years). Women predominated, comprising 252 of the 373 patients, which represents a sex ratio of 67%.

One study did not specify the sex of the patients [28].

- Anatomical localization

Of the articles included for which the location was specified, 91 patients had involvement of the lower limb, including 25 cases involving the hallux when this information was available, and 164 patients had involvement of the upper limb, including 50 cases involving the thumb. However, for 118 patients, the precise anatomical location was not reported. Four studies did not mention the location of the lesion at all [17,19,24,27].

- Melanoma type

Regarding the diagnostic phase, the majority of articles [18,22,24,25,26,28,30,32,33,35,36,37] reported that an initial biopsy was performed. Only one article [34] mentioned an immediate biopsy-exeresis, while two studies [20,38] noted the absence of a biopsy. The presence or absence of prior biopsy was not specified in nine articles [15,16,17,19,21,23,27,29,31]. When the degree of local invasion was reported, the majority of melanomas were in situ (*n* = 65). Among the invasive forms, five cases had a Breslow index of less than 1 mm, two cases had a Breslow index between 1 and 3 mm, and two cases had a Breslow index of more than 3 mm. Six studies did not mention the stage of the melanoma [19,20,22,23,24].

- Margins

These margins were not tumor resection margins in the strict sense of the term, but margins for resection of the entire nail apparatus, generally set at 5 mm in the majority of studies [15,16,17,18,19,20,21,24,26,36,37,38]. However, some publications have reported wider margins of 10 mm [25,33,35,37], while others have recommended narrower margins of 3 mm [22,34]. The standard Mohs micrographic surgery technique was used in two studies [27,32], and the delayed Mohs or ‘Slow Mohs’ technique was also mentioned in two other publications [27,32]. Finally, one study reported the use of extemporaneous anatomopathological examination to check the margins of excision of the nail apparatus [30].

- Strategy for reconstruction

Full-thickness skin graft (FTSG) was the preferred reconstruction technique in the majority of studies [15,16,17,18,19,20,21,23,25,26,29,32,33,34], involving a total of 112 patients. Of these, ten studies [16,17,18,21,25,26,29,32,33,34] reported that FTSG was performed immediately, at the same time as the extended excision, in 52 patients. In contrast, seven studies reported delayed reconstruction, involving 55 patients [16,17,19,20,26,30,31,32,34,35,38]. The immediate or delayed nature of the reconstruction was not specified in four publications [15,20,23,27].

Only the series by Crisan et al. reported the use of a negative pressure dressing in preparation for delayed FTSG [31]. FTSG was used exclusively in 71 patients [15,16,17,20,21,23,25,26,31,32,33,34], combined with a dermal matrix in 5 cases [30,35,38], and combined with a perionychium flap in 43 patients [18,19,21]. One study used a delayed thin skin graft after Mohs micrographic analysis [27].

Concerning the use of dermal matrices, three articles [30,35,38] were concerned, involving a total of five patients, for whom the dermal matrix was placed at the same time as the resection, and a second stage with the placement of an FTSG three weeks after the first stage was carried out. Only the PELNAC^®^ matrix was used, except in one study where INTEGRA^®^ was used [35].

With regard to flaps, only Motta et al. and Lee et al. [24,37] utilized free flaps, respectively, a free toe transfer in 1 case, and ultra-thin superficial circumflex iliac artery perforator flaps (SCIP) in 41 cases. In contrast, eight studies mentioned local flaps, six of which were associated with an FTSG [15,18,19,20,21,32] and two used exclusive Foucher flap reconstruction [28,36]. Local flaps combined with FTSG were most often perionychium advancement or palmar advancement flaps [18,19,21]; one case report proposed a cross-finger flap [32]. Finally, two studies favored directed healing only [20,27], and one study did not mention the surgical technique for reconstruction [37].

- Carcinologic complications

The most frequently observed complication was melanoma recurrence, which was reported in 11 studies [17,19,20,21,22,23,24,26,27,28,31,32] involving a total of 67 patients. Of these, 13 required secondary amputation of the affected digit [17,20,21,26,27,28,31,32]. In two cases, recurrence was linked to histologically positive excision margins, requiring amputation [17,31].

In a further 33 cases, the status of the margins was not specified, and the treatment options varied: 11 patients underwent amputation [20,21,26,27,28,32], and 5 underwent revision surgery of the margins [19,24,27], including 3 by deferred Mohs technique [27].

Early recurrences, occurring within the first year after surgery, were reported in 15 patients [17,19,20]. There were two cases of SUM in situ and one invasive SUM with a thickness of 2.5 mm when specified [19,22,30]. The margins used in these studies were 3 mm in the series by Oh et al. [22], 5 mm in two series [17,19], and between 5 and 10 mm in one series [22]. The mean time from diagnosis to occurrence was 8 months after surgery.

Late recurrences, defined as occurring at least one year after surgery, were the most frequent, with 28 patients reported [18,20,21,22,24,25,30]. These included SUM in situ in five cases [24,25], invasive stage T1 in one case [25], stage T2 in two cases [22,25], stage T3 in two cases [25], and stage T4 in two cases [21,25], as specified. Among these articles, the margins used around the nail apparatus were 3 mm [20], 5 mm [18], 6 mm [24,25], and 5 to 10 mm [22]. These recurrences were found at the surgical site in 36 cases [18,20,24,25], in transit in 1 case [22], and reached the lymph node area concerned in 1 case [21]. The average time to recurrence was 29 months after surgery.

As regards distant localization recurrence, 21 cases were described [19,21,22,23,24,27], including the following:

○ Two transit metastases, one of which was treated surgically [19,24];
○ Ten lymph node involvement [19,23];
○ Eleven visceral metastases, resulting in 2 deaths [19,21,22,27].

These recurrences occurred on average 20 months after the first operation. They complicated a grade T3 melanoma in five cases [1,25] and a grade T4 in one case [19]. The nail margins found in these cases measured 5 mm in three series, involving 18 patients [17,19,25].

- Non-carcinologic post-operative complications
  ○ Residual nail spicules were reported in 15 patients [15,17,18,20], and epidermal cysts were found in 13 patients [15,16,17,20]. Other adverse events included the following:
  ○ Ten cases of hypersensitivity to cold or shock;
  ○ Nine cases of graft hyperpigmentation [15];
  ○ One case of distal interphalangeal arthritis [31];
  ○ One flexor sheath hematoma [26];
  ○ One case of temporary functional exclusion of the finger [16].

Six studies reported no complications [25,29,30,35,36,38], and two did not mention this criterion [33,37]

- Complementary treatments

Most studies did not mention complementary treatments (*n* = 23).

Only one study (31) described complementary treatments, with an adjuvant course of Interferon alpha for one patient, in the context of the appearance of distant metastases. This same study also described a case of recurrence treated by radiotherapy, with an unspecified impact on the outcome of reconstruction, and a case of multiple recurrences of metastases in transit treated by iterative excision and directed healing. The sentinel node procedure was mentioned only in the study by Wollina et al., involving four patients, only one of whom had a micrometastasis [23]. No study mentioned immunotherapy.

- Functional and cosmetic results

Post-operative functional and cosmetic results were assessed by the examiner in six studies [20,31,33,34,35,37], by the patient in six other studies [16,17,18,24,30,36], and by the patient and the examiner in one study [25]; six studies did not mention who the assessor was [15,19,26,27,29,35,38].

Functional recovery was judged acceptable to satisfactory in eight studies, involving a total of 80 patients [15,16,18,19,20,25,30,31], whereas it was considered total, with a return to normal function, in seven studies [16,20,26,34,35,36,37] involving 39 patients.

Regarding the cosmetic result, it was judged to be little disturbed or good in 12 articles [15,17,18,19,20,25,28,30,31,33,35,38], involving 76 patients. In contrast, it was judged to be excellent or equivalent to the previous state in four studies [20,26,36,37], involving 42 patients with heterogeneous surgical techniques: exclusive directed healing [20], Foucher flap in the case report of one patient [36], free onycho-cutaneous flap harvested from the hallux in the case report of one patient [37], and six cases treated by FTSG and five by exclusive directed healing in the series by Neczyporenko et al. [26]. The cosmetic result was judged to be poor in only two patients in the series by Goettmann et al. [20], but although directed healing was the predominant choice, the technique used for these two patients was not specified.

Only one article classified its results using validated questionnaires, that of Lee et al. [24], which found an average Quick-Disabilities of the Arm, Shoulder and Hand (DASH) score of 1.3 in the 14 patients undergoing surgery on the upper limb and an average Foot Function Index (FFI) score of 3.1 in the 12 patients undergoing surgery on the lower limb.

The time to return to work was specified in two studies [31,35], and averaged four and six weeks, respectively, after the last operation.

- Post-operative follow-up

Reported follow-up ranged from 3 months [36,37,38] to 120 months [20].

The mean follow-up was 60.1 months, with a mean of 45 months.

## 4. Discussion

The management of subungual melanoma has evolved significantly since its first description in the nineteenth century [39]. This topographical form appears to affect women preferentially, as indicated by our review (women authored 67% of the 24 articles included), in line with the data of Neczyporenko et al. [26].

Regarding the age of onset, the fifth decade is the most frequently reported, with a few exceptions, such as the series by Lazar et al. [16], where the mean age was 40. Preoperative biopsy is not usually recommended for pigmented skin lesions in favor of excisional biopsy [40]. However, in certain functional or aesthetically sensitive areas, a biopsy may be justified as a first-line procedure. In our review, several studies did not specify the performance of a biopsy and its modality, or mentioned the absence of one, which exposes the risk of unnecessary mutilating surgery, even in the presence of effective reconstruction techniques. EAU is now preferred to amputation in cases of in situ or minimally invasive melanoma, although there is no agreed Breslow threshold [41,42]. Most articles support this strategy, with a recurrence rate comparable to that of amputation [16,19,28,43,44].

Although some studies report more local recurrences after WLE, these often occur after the operation. However, it should be noted that this technique is primarily intended for less aggressive tumors without metastatic disease. Furthermore, anatomopathological control may be incomplete, as pointed out by Kimyai-Asaidi et al. [45]. This also explains why sentinel lymph nodes are rarely used in these cases. Conversely, amputations are reserved for more advanced forms, with a higher metastatic potential.

There is no clear consensus on whether to perform immediate versus delayed reconstruction. Our literature review revealed a notable rate of local recurrence, with 67 out of 373 patients (approximately 18%) experiencing recurrence, most often requiring secondary amputation. When reported, the time to recurrence ranged from 5 months to 11 years. Unfortunately, the recurrence details were not consistently reported by the authors. The timing of recurrence was mentioned in only two studies: at 24 and 32 months in Goettmann et al. [20] and at 36 months in Rayatt et al. [28]. Due to the heterogeneity in follow-up protocols and limited data, it was difficult to establish a clear correlation between surgical margin size and recurrence rate. However, the most extensive patient series, comprising 140 cases and reporting a recurrence rate of 16.4%, used low margins of 3 to 4 mm, including periosteum [20]. Reported cases of amputation following histological analysis of the resection specimen—including two in the literature—further support the rationale for delayed reconstruction, particularly in cases of invasive melanoma.

Two techniques have been proposed for margin assessment: intraoperative frozen section analysis, which is limited by the absence of specific immunostains (such as MART-1 and HMB-45), and the Mohs micrographic surgery technique, which can be performed either conventionally or in a staged manner. While Mohs is not recommended as a standard treatment for melanoma, it is employed in certain specific cases, particularly for lentigo maligna (Dubreuilh’s melanoma) [6,45,46,47]. It allows more precise histological control, but its accessibility remains limited, and its efficacy in this context is still debated. Most articles support this strategy, with a recurrence rate comparable to that of amputation [16,19,28,42,43]. Although some studies report a higher rate of local recurrence after wide local excision (WLE), it is important to note that this technique is generally reserved for less aggressive tumors without metastatic spread, which may influence recurrence patterns observed postoperatively.

Furthermore, anatomopathological control may be incomplete, as noted by Kimyai-Asaidi et al. [45]. This also explains why sentinel lymph nodes are rarely used in these cases. Conversely, amputations are reserved for more advanced forms, with a higher metastatic potential.

Reconstruction approaches vary from total skin grafts, dermal matrices, to pedicled or free flaps [17,22,26]. The choice depends on the location, the functional importance of the finger or toe, the need for mechanical strength, or, on the contrary, aesthetic requirements. Free flaps or flaps with a temporary pedicle (flag flap type) may be too invasive for certain indications [26]. In contrast, a graft combined with a dermal matrix is often a good compromise, especially for distal injuries with poor function [28]. The inner arm is frequently used as a donor site, due to its hairless and discreet appearance [48]. Dermal matrices provide post-operative comfort (reduced hypersensitivity) and allow coverage of exposed bone, enabling WLE with sub-periosteal resection or removal of the dorsal cortex of the last phalanx; however, they require additional operating time [28,33,36]. For toe lesions, healing by secondary intention can also be considered, as it is often well tolerated when wearing protective footwear. These approaches remain eligible for outpatient surgery under locoregional anesthesia.

In our review, most of the reconstruction techniques [13,14,15,16,17,18,19,21,23,24,27,30,31,32] described were based on skin grafting, alone or in association with a matrix or local flap, which could be explained by the low logistical and organizational constraints of this procedure, the quality of the results it provides, its simplicity of execution and its independence in relation to the size of the recipient site. Although a single surgical procedure is the most ergonomic option, our review found a similar number of one-stage and two-stage procedures. The latter, sometimes due to the time required to incorporate the matrix, could be dispensed with by the advent of single-layer matrices. As for the functional and cosmetic results mentioned in our review, they were often judged as “good”, “acceptable”, or “satisfactory”, which unfortunately made interpretation difficult and subjective. Objective results, at best reported by the patient using validated scores, would enable an effective comparison of the different techniques for a given patient profile.

In our experience, reconstruction should be adapted as far as possible to the patient’s profile [49,50]. However, for upper limb localization, the combination of a dermal matrix and a total skin graft appears to us to be the most suitable technique, due to the high quality of the functional and cosmetic results. For lower limb injuries, the use of pedicled flaps, such as the intermetatarsal flap [51], is our first-line option for reconstruction.

Neoadjuvant immunotherapy, already used for resectable stage III melanoma, could represent a turning point for localized forms such as SUM [52,53]. In the long term, it could make it possible to avoid certain amputations in favor of more conservative surgery such as WLE. However, these therapeutic options have not been widely studied, reflecting the persistent compartmentalization between medical and surgical disciplines.

The main limitations of our review relate to the lack of standardized functional evaluation of reconstructions, the heterogeneity of surgical techniques, and the absence of precise data on histological stage and recurrence. Overall study quality, as assessed using the Newcastle–Ottawa Scale and Joanna Briggs Institute checklists, was moderate: inclusion criteria and surgical protocols were generally well described, but sample sizes were small, techniques varied widely, and comparative analyses were scarce. Functional and aesthetic outcomes were assessed in a largely non-standardized manner, most often based on qualitative appraisal (surgeon judgment, patient-reported satisfaction or pain, or return to usual activities). Only Lee et al. [24] employed validated PROMs (QuickDASH and Foot Function Index), reporting superior functional scores compared with amputation. This heterogeneity and lack of standardized tools limit comparability across studies and may overestimate the functional and aesthetic benefits of conservative approaches. Nevertheless, most series reported preserved function and satisfactory cosmetic outcomes after functional surgery. Future prospective multicenter studies should systematically use validated PROMs (QuickDASH, Foot Function Index, POSAS, SCAR-Q) and standardized definitions for local and distant recurrence to more accurately assess the oncological safety and long-term quality-of-life impact of reconstructive techniques, particularly in thin invasive lesions.

## 5. Conclusions

The management of subungual melanoma is increasingly shifting towards WLE rather than amputation, particularly for in situ lesions or those with a low Breslow index. Across the included series, the recurrence rate is approximately 18%, with recurrence occurring between 5 months and 11 years, highlighting the need for long-term, systematic follow-up. Reconstructive techniques such as total skin grafts or dermal matrices can achieve satisfactory functional and aesthetic outcomes, yet objective, standardized evaluations are scarce, and adjuvant therapies are rarely reported. Future prospective, multicenter studies with standardized oncologic, functional, and quality-of-life endpoints are essential to optimize treatment strategies and ensure durable, patient-centered outcomes.

## Figures and Tables

**Table 1:** Characteristics of included studies on reconstruction after wide local excision for subungual melanoma.

Ref.	Type of Article Number of Patients Distribution Mean Age (Years) (Range)	Localization F (Finger) T (Toe) (n)	Local Extension	Presence of Previous Biopsy Margins from Nail Apparatus	Strategy of Reconstruction Surgical Management	Side Effects, Complications	Complementary Treatment	Results	Follow Up Mean (Range)
Anda-Juarez et al., An Bras Dermatol, 2016 [15]	Retrospective 15 patients (9F, 6M) 31 (4–66)	Right hand: F2 (2), F4 (2), F5 (1) Left hand: F1 (1), F5 (3) Right foot (5): T1 (5) Left foot (1): T1	SUM in situ	5 mm Supra periosteum resection	FTSG (14) Banner flap (1 patient)	No recurrence Inclusion cysts (4) Spicules (6) Hypersensitivity (4) Moderate chronic pain (1) Hyperpigmentation of the skin graft (9)	NA	Functional and cosmetic outcomes were good in all of them	55 months (12–98)
Lazar et al., HandSurg Br, 2005 [16]	Retrospective 13 patients (8F, 5M) 10 patients with SUM (7F, 3M) 40 (NA)	Right hand F1 (3), F3 (1), F5 (1) Left hand: F1 (2), F5(3)	SUM in situ	5 mm	Immediate FTSG harvested from same forearm (9) Delayed FTSG harvested from same forearm (1)	Temporary finger exclusion (1 case) Epidermal cyst (5 cases)	NA	Sensitivity: Weber 4–6 mm (7), NA (1) Normal function (4), Slightly limited function (5), NA (1) Cosmetic outcome: Satisfied or normal (9), NA (1)	48 months (6–84)
Puhaindran et al., Tech Hand Up Extrem Surg, 2011 [17]	Retrospective 10 patients (4F, 6 M) 6 patients with a SUM MA 52 (24–80)	NA	SUM in situ, <2 cm diameter	2 mm to nail fold	Immediate FTSG	Positive margin (1), treated by PID Disarticulation Secondary surgery: Epidermal inclusion cyst (1) Nail remnant (1)	NA	Acceptable appearance for all patients (assessed by 2 surgeons) Patients satisfied (6) Unrestricted use of the hand (6)	35 months (8–72)
Flores-Terry et al., Actas Dermosifiliogr, 2018 [18]	Retrospective 11 patients 7 patients with SUM (4F, 3M) 5 patients (3F, 2M) treated by WLE 61 (45–81)	Right hand: F2 (1) F3 (1) Left hand F1 (1) F2 (1) Right foot: T1 (1)	SUM in situ (4) or Sum with Breslow < 1 mm (1)	Previous biopsy 5 mm Supra periosteum resection	Circumferential advancement flap and Immediate FTSG	No recurrency (5) Wound infection (2) subungual spicules (1), moderate stiffness of DIP of one finger (1), hypersensitivity to cold (2), hypersensitivity to mild trauma (4)	NA	Patients were satisfied with the procedure and the results obtained (5) Satisfaction was good, and the impact on quality of life was minimal (5)	39 months (12–96)
Moehrle et al., Dermatol Surg, 2003 [19]	Retrospective 62 patients with SUM (25H, 37F) 31 treated by WLE (11H, 20F) MA 61	F1,2,3,4,5 (20) T1,2,3,4,5 (11)	SUM in situ Invasive SUM Breslow < 1 mm (6) Breslow 1 to 2 mm (8) Breslow 2 to 4 mm (6) Breslow > 4 mm (4) NA: (7)	5 mm WLE with safety margin without bone resection (3) WLE with safety margin with resection of the distal part of the distal phalanx (28)	FTSG with pulpal advancement flap Single stage (1) Several stages (30) Unspecified reconstruction after definitive three-dimensional histology	Recurrences (20 patients) Local recurrence (2) In transit recurrence (1) Lymph node metastasis (7) Distant metastasis (1)	NA	Function and cosmesis of the involved finger or toe “preserved”	54 months (NA)
Goettmann et al., J Eur Acad Dermatol Venereol, 2018 [20]	Retrospective 63 patients (44F, 19M) 58 treated by WLE 51 (NA)	F1 (24) F2,3,4,5 (23) T1 (10) T3 (1)	All SUM, if pulp is not involved	No previous biopsy WLE (12) Partial excision of the appliance, removal of the lesion, and the nearby paronychium (47)	Healing by secondary intention (52) FTSG (3 patients) Flap (1)	Local recurrences at 24 and 32 months treated by amputation (2) Spicules (7) Epidermal cysts (2)	NA	No functional discomfort (20) Moderate discomfort (14) Aesthetic discomfort was judged to be absent (29) Moderate aesthetic discomfort (8) Severe aesthetic discomfort (2)	120 months (NA)
Chow et al., J Plast Reconstru Surg, 2013 [33]	Case report 1M with SUM in situ 41	Right T1 (1)	SUM in situ	Biopsy 10 mm WLE with a layer of bone	Immediate FTSG (harvested from the groin)	NA	NA	Acceptable cosmetic result (assessed by surgeon)	5 months
Duarte et al., Dermatol, 2010 [34]	Case report 1F with SUM in situ 61	Right F1 (1)	SUM in situ	Excisional biopsy 3 weeks later 3 mm margin WLE	FTSG taken from the arm	No local recurrence or metastasis	NA	Thumb function completely preserved	12 months
High et al., Arch Dermatol, 2004 [32]	Retrospective 7 patients (5F, 2M) with SUM in situ 4 patients treated by WLE MA 56 (NA)	Right hand (1): F2 Left hand (2): F2 (1), F5 (1) Right foot (1): T1 (1)	SUM in situ	Previous biopsy MOHS surgery: 1 stage (3) 2 stages (1)	FTSG taken from the arm (3) cross finger flap (1)	Recurrence: (1) treated by revisional amputation	NA	NA	24 months (10–29)
Sinno et al., J Plast Surg Hand Surg, 2015 [21]	Retrospective 35 patients with melanoma of the hand (24F, 11M) 18 patients with SUM 10 patients with SUM treated by WLE	F1 (8) F2 (12) F3 (3) F5 (3)	Melanomas in situ (7) Invasive Melanoma T2 (1 patients) B = 2.5 mm Invasive Melanoma T3 (2); B = 3.00 mm and B = 3.08 mm	5 mm	FTSG (3) Paronychial advancement flap + FTSG (3) Paronychial advancement flap + forearm flap + FTSG (1) Paronychial advancement flap + FTSG Volar Flap FTSG (1) FTSG Volar and dorsal advancement flaps (1) FTSG Local advancement flap (1)	Revisional amputation (3) Unknown (1) Deceased (1)	NA	NA	47 months (7–74)
Smock et al., J Plast Reconstr Aesthet Surg, 2010 [35]	Case report 1M 44	Right F1 (1)	Ulcerated SUM Breslow of 1.2 mm	Previous Biopsy 10 mm, including the periosteum	Immediate reconstruction by dermal matrix (INTEGRA) STSG 3 weeks later	None	NA	Fully functional thumb and a good cosmetic result Went back to job at 4 weeks	24 months
Bjedov et al., Acta Dermato Venereol Croat, 2019 [36]	Case report (1) F, 31	Left F1 (1)	SUM in situ	Several nail matrix biopsies 5 mm, with periosteum Mohs analysis	Pedicled innervated Fascio-cutaneous Foucher’s flap The donor site was covered with an FTSG taken from the volar side of the elbow.	None	NA	The hand was fully functional, and the patient was very satisfied with the appearance of the thumb Full sensory cortical reorientation	3 months
Oh et al., J Am Acad Dermatol, 2023 [22]	Retrospective 140 patients with SUM 107 with conservative treatment (57F, 50H) Mean age: 56	F1,2,3,4,5 (71) T1,2,3,4,5 (36)	If no bone invasion (MRI + biopsy)	Biopsy and MRI at least 3 to 4 mm, with periosteum	NA	Recurrence (23 patients): Local recurrence (15) Distant recurrences (8)	NA	NA	45 months (14–76)
Motta et al., Arch Dermatol, 2007 [37]	Case report 12 years old, F	Right F1 (1)	SUM in situ	Biopsy WLE with Mohs Micrographic analysis 5–10 mm (lateral) 5 mm (proximal and distal edges)	Two stages Second stage (1 week later) microvascular composite onychocutaneous free flap from the right first toe	NA	NA	Normal nail growth and full mobility of the interphalangeal thumb joint were present	3 months
Wollina et al., Dermatol Ther, 2019 [23]	Retrospective Series of 12 patients with SUM) 6 patients with conservative treatment (2F, 4M) 76 (NA)	Right foot T1 (2) T3 (1) Left foot: T1 (3)	SUM with Breslow between 1.6 mm and 4.8 mm	Wide excision (4) Excision with delayed Mohs surgery (2)	Full-thickness skin transplantation. One patient refused, second intention healing	Local relapse (2) Later metastasis (1) Satellites only (1 patient, B = 2.55) Liver, pancreas, spleen, lymph nodes, stomach, adrenal glands, greater omentum, CNS (1, with Breslow = 4.8 mm) In transit, lymph node regional, pericardium (1, with Breslow = 3.20)	Sentinel Lymph node (4), with micro-invasion (1) Polychemotherapy for later metastasis (1) Transit metastases treated by erbium YAG-laser as a palliative measure (1) interferon-alfa therapy for 9 years after surgery, satellite metastasis (1) Adjuvant radiotherapy (1)	NA	104 months (17–208)
Crisan et al., Acad Dermatol Venereol, 2017 [31]	Retrospective Series of 7 patients (3F, 4H) 64 (NA)	F1 (3) F4 (3) T1 (1)	SUM in situ pT1a (1) pT1b (1)	WLE (7) NA	First stage: vacuum-assisted closure Second stage: FTSG (5)	Second stage amputation (2 patients) for: -DIP joint arthritis (1) -Positive margin (1)	NA	At 6 weeks, the 5 patients grafted could resume normal activity Good cosmetic and functional	12 months (NA)
Lee et al., Plast Reconstr Surg, 2017 [24]	Prospective 41 patients with conservative treatment (21M, 20F) 51.1 (NA)	Fingers: 25 Toes: 16	SUM with Breslow thickness of ≤2 mm on preoperative biopsy	Preoperative biopsy 5 mm with periosteum excised (10 mm margin if invasive lesion)	Immediate reconstruction by SCIP flap with a final thickness ranging from 1.5 to 4 mm after defatting (Scouted with Doppler) End-to-end anastomosis with digital artery and dorsal vein Donor site primary closing	Necrosis of the flap (1) (arterial insufficiency) Venous congestion (3) with partial necrosis of the flap Seroma of the donor site (1). Recurrences: -local recurrence (1) -metastasis in transit (1) Second surgical stage until degreasing (12)	NA	Average healing time: 15 days Questionnaire carried out on 26 patients: WLE in the upper limb (14): The mean Quick-DASH score was 1.3 (range 0 to 6.8). WLE in the lower limb (12): FFI survey for foot lesions. mean score was 3.1 (range 0 to 8.0)	31 months (NA)
Hayashi et al., Dermatol Surg, 2012 [38]	Case report (1M), 52	Left F3	SUM in situ	No biopsy 5 mm with excision of periosteum	Immediate reconstruction with artificial dermis (PELNAC) Second stage at 4 weeks with FTSG	No recurrency No metastasis	NA	Good cosmetic results	3 months
Sureda et al., Br J Dermatol, 2011 [25]	Retrospective Series of 7 patients (5F, 2M) MA 58 y	F1 (2), F2 (2), F4 (1) T1 (2)	SUM in situ (5) or minimally invasive SUM (2) (Breslow 0.2 and 0.15)	Biopsy systematically 5–10 mm Deep margin was bone contact	Immediate FTSG taken from the internal aspect of an arm	No recurrence	NA	Interrogation of patient and observer: High level of satisfaction, a good functional and quite good cosmetic result.	45 months (24–84)
Neczyporenko et al., J Eur Acad Dermatol Venereol, 2014 [26]	Retrospective Series of 11 patients (8F, 3M) 48 (NA)	Right hand: F1 (3), F2 (1), F5 (1) Left hand: F1 (1) F2 (1) T1 (3), T2 (1)	Melanoma in situ	Biopsy systematically (tangential or punch) 6 mm	Immediate FTSG (6) Secondary intention and delayed FTSG (5)	Tendon sheath hematoma (1) Lymphangitis post (1) Recurrence treated by secondary amputation and sentinel node (2) (7- and 11 years post op)	NA	Healing by secondary intention and grafting were fully satisfactory, cosmetically and functionally	65 months (5–167)
Terushkin et al., Dermatol Surg, 2016 [27]	Retrospective Series of 40 patients (21F, 19M) 63 (NA)	NA	WLE in cases of extensive SUM (>40% of the bed)	Excisional biopsy with Mohs micrograph, Supra Periosteum dissection	Second intention FTSG STSG	Recurrences (5) Second operation with complete excision of the matrix (2) Amputation (2) Death due to metastasis (1)	NA	NA	76 months (2–276)
Rayatt et al., Plast Reconstr Aesthet Surg, 2007 [28]	Retrospective Series of 4 patients 56 (NA)	F1 (4)	SUM without deep margin clinically involved Breslow 0.9 mm to 4 mm	All had initial biopsies to confirm the diagnosis 10 mm including the periosteum	Immediate: -Foucher flap (1) -Flag flap (2) Delayed reconstruction (flag flap) (1)	Recurrence at 36 months treated by amputation (1)	NA	Usefully maintain function.	72 months (−117)
Imakado et al., J Dermatol, 2008 [29]	Retrospective Series of 2 patients (1M, 1F) 50	Left F3 (1) Right F5 (1)	SUM in situ	All the nail apparatus with nail folds	Immediate FTSG	No recurrence	NA	NA	27 months (6–48)
Liu et al., Medicina, 2020 [30]	Retrospective Series of 4 patients with malignant tumor of nail apparatus 3 patients with SUM suspicion (1F, 2M) MA 55 y	Left T1 (1) Right T1 (2)	SUM in situ (2) hyperpigmentation in basal layer of epidermis (1)	Previous biopsy (2) WLE Adequate margin control confirmed by intraoperative frozen sections	Two-stage reconstruction Immediate acellular dermal matrix (PELNAC) At 10 days, the acellular dermal matrix was removed and re-dressed with a new one at the outpatient clinic FTSG at 3 weeks	No Recurrence	NA	These patients experienced minimal change in body contour, mild but acceptable functional deficit, and satisfying aesthetic results.	9 months (5–13)

## Data Availability

The corresponding authors can provide the data upon request.

## Supplementary Materials

The following supporting information can be downloaded at: https://www.mdpi.com/article/10.3390/jcm14175932/s1, Table S1: Quality assessment of included studies according to the Newcastle–Ottawa Scale (NOS) or the Joanna Briggs Institute (JBI) checklist, depending on study design. “Instrumented validates” refers to the use of standardized, psychometrically validated tools.

## Abbreviations

The following abbreviations are used in this manuscript:

SUM	Subungual melanoma
WLE	Wide local excision (of nail apparatus)
F	Finger
T	Toe
FTSG	Full-thickness skin graft
STSG	Split-thickness skin graft
NA	Non available
Quick DASH	Score Quick Disabilities of the arm, shoulder, and hand (score ranges from 0, no obstacles, to 100, maximum obstacles)
MMWS	score ranging from 0, maximum disability, to 100, no disability
Score AOFAS	The American Orthopedic Foot and Ankle Score (score ranging from 0, maximum disability, to 90, no disability)
FS	Functional surgery
NOS	Newcastle–Ottawa Scale
JBI	Joanna Briggs Institute checklist
FFI	Foot Function Index
PROMs	Patient-reported outcome measures
SCC	Squamous cell carcinoma
