# Supplementary material for: Reconstruction After Wide Excision of the Nail Apparatus in the Treatment of Melanoma: A Systematic Literature Review
Source: J Clin Med. 2025 Aug 22;14(17):5932. doi: 10.3390/jcm14175932 (PMC12429834; doi:10.3390/jcm14175932)
Supplement: Supplementary file 1 [file jcm-14-05932-s001.zip › jcm-3802021-supplementary.pdf]

Ref.	Citation (short)	Design	N (pts/digits)	Quality tool	Quality rating	Functional/Cosmetic assessment method	Validated instrument?
15	De Anda-Juárez 2016, An Bras Dermatol	Retrospective case series (in situ SUM)	15	NOS (adapted for case series)	Moderate	Clinical review + patient-reported pain and sensitivity	No
16	Lazar 2005, J Hand Surg Br	Retrospective technique/series (nail unit recon)	13 (mixed nail tumors incl. in situ)	JBI (case series)	Moderate	Examiner assessment incl. sensibility (Weber 2-point), return to normal/slightly limited function, patient cosmetic satisfaction	No (clinical scales)
17	Puhaindran 2011, Tech Hand Up Extrem Surg	Retrospective review (melanoma in situ/early SCC)	9 pts / 10 digits	NOS	Moderate	Return to presurgery hand use; patient satisfaction documented	No
18	Flores-Terry 2018, Actas Dermosifiliogr	Retrospective series + literature review	11 tumors (7 SUM, 4 SCC)	NOS	Moderate-High	Post-op survey (Likert) + charted complications	No (Likert)
19	Moehrle 2003, Dermatol Surg	Retrospective comparative (FS vs amputation)	62	NOS (comparative cohort)	Moderate-High	Qualitative reporting of function and cosmesis	No
20	Goettmann 2018, JEADV	Retrospective cohort (SUM in situ)	63	NOS	High	Patient-reported sensitivity/dexterity (chart extraction) + clinician assessment	No
21	Sinno 2015, J Plast Surg Hand Surg	Retrospective review + algorithm	35 (subset SUM)	JBI (heterogeneous series)	Moderate	Not systematically reported	No
22	Oh 2023, JAAD	Retrospective cohort (FS vs amputation)	140 (107 FS; 33 amp)	NOS	High	Not the focus; recurrence/factors primary endpoints	No
23	Wollina 2019, Dermatol Ther	Retrospective single-center series	12	NOS	Moderate	Not reported	No
24	Lee 2017, Plast Reconstr Surg	Prospective case series (SCIP flap)	41 (≤2 mm Breslow)	NOS (prospective series)	Moderate-High	Validated PROMs: QuickDASH (hand) & Foot Function Index (foot)	Yes (QuickDASH, FFI)
25	Sureda 2011, Br J Dermatol	Retrospective series + literature review	7 + review	NOS	Moderate	Patient & physician satisfaction (qualitative)	No
26	Neczyporenko 2014, JEADV	Retrospective series (SUM in situ)	11	NOS	Moderate-High	Cosmetic/functional acceptability + complications documented	No
27	Terushkin 2016, Dermatol Surg	Retrospective MMS series (digital melanoma)	62 (40 nail unit)	NOS	High	Amputation avoidance, local control; no PROMs	No
28	Rayatt 2007, JPRAS	Small case series (thumb SUM)	4	JBI (case series)	Moderate	Return to work; qualitative functional assessment	No
29	Imakado 2008, J Dermatol	Case reports (2)	2	JBI (case reports)	Low (case level)	Narrative clinical assessment	No
30	Liu 2020, Medicina (Kaunas)	Small series (reconstruction with ADM→FTSG)	4	JBI (case series)	Moderate-Low	Clinical take rate; patient-reported aesthetic satisfaction	No
31	Crisan 2017, JEADV (Letter)	Case series (digit-preserving VAC→FTSG)	7	JBI (case series)	Moderate-Low	Clinical assessment; return to activity	No
32	High 2004, Arch Dermatol	Case series (SUM in situ nail unit; MMS)	7	JBI (case series)	Moderate	Amputation avoidance + clinical course	No
33	Chow 2013, JPRAS	Case report (digit-salvaging clearance)	1	JBI (case report)	Low (case level)	Surgeon-rated cosmetic acceptability at 5 months	No
34	Duarte 2010, Dermatology	Case report (matrix in situ melanoma)	1	JBI (case report)	Low (case level)	Clinical: functional preservation at 12 months	No
35	Smock 2010, JPRAS	Case report (Integra → STSG)	1	JBI (case report)	Low (case level)	Clinical narrative; return to work timepoint	No
36	Bjedov 2019, Acta Derm Croat	Case report (Foucher flap)	1	JBI (case report)	Low (case level)	Patient satisfaction + clinical function and sensation	No
37	Motta 2007, Arch Dermatol	Case report (pediatric; slow Mohs + free toe onych	1	JBI (case report)	Low (case level)	Clinical: normal nail growth; joint mobility	No
38	Hayashi 2012, Dermatol Surg	Case report (artificial dermis → FTSG)	1	JBI (case report)	Low (case level)	Clinical photographic assessment; cosmesis at 3 months	No

Supplementary Table S1. Quality assessment of included studies according to the Newcastle–Ottawa Scale (NOS) or the Joanna Briggs Institute (JBI) checklist, depending on study design. “Instrumented validates” refers to the use of standardized, psychometrically validated tools.

Notes on outcomes
Good function overall; 1 mild persistent pain; frequent nail spicules/inclusion cysts reported
Most satisfied cosmesis; some epidermal cysts; temporary functional exclusion in 1
All satisfied; all returned to presurgery use; no local recurrences reported
High satisfaction; preserved function; no local/regional recurrences in WLE group
Better functional/cosmetic with FS; no survival disadvantage vs amputation
Generally satisfactory; 2 late in situ recurrences over ~10-yr mean follow-up
Narrative assessment of safety of conservative excision; varied recon options
Breslow ≥0.8 mm associated with higher recurrence; FS reasonable for Breslow <0.8 mm
3D margin control; delays common; survival data provided
Significantly better function vs amputation controls; 3-yr DFS 97.1%
Satisfactory function/cosmesis; no recurrences
Satisfactory; 2 very late local recurrences (7 & 11 yrs)
Local recurrence 8.2%; 0% with MART-1 immunostaining; 96.5% digit-sparing
Length preserved; minimal disability; 1 local recurrence → amputation
Acceptable outcomes; no recurrence reported in follow-up
Excellent graft take; satisfying aesthetics; short follow-up
Good cosmetic/functional; short follow-up; no local recurrences
No recurrences; most digits preserved
Acceptable cosmesis; FTSG over shallow cortical bone resection
Thumb function completely preserved; good cosmesis
Fully functional thumb; good cosmetic result; back to work after graft check
Fully functional; innervated flap; high satisfaction at 3 months
Normal nail growth and full mobility at 3 months
Good cosmetic result; 2-yr disease-free follow-up
